# A novel walking speed estimation scheme and its application to treadmill control for gait rehabilitation

**DOI:** 10.1186/1743-0003-9-62

**Published:** 2012-08-28

**Authors:** Jungwon Yoon, Hyung-Soon Park, Diane Louise Damiano

**Affiliations:** 1 Rehabilitation Medicine Department, Clinical Center, National Institutes of Health, Bethesda, MD, USA; 2 School of Mechanical Engineering & ReCAPT, Gyeongsang National University, Jinju, Republic of Korea

**Keywords:** Body-weight supported treadmill training, Walking velocity estimation, Self-selected treadmill speed control, Gait analysis, Temporal-spatial gait parameters

## Abstract

**Background:**

Virtual reality (VR) technology along with treadmill training (TT) can effectively provide goal-oriented practice and promote improved motor learning in patients with neurological disorders. Moreover, the VR + TT scheme may enhance cognitive engagement for more effective gait rehabilitation and greater transfer to over ground walking. For this purpose, we developed an individualized treadmill controller with a novel speed estimation scheme using swing foot velocity, which can enable user-driven treadmill walking (UDW) to more closely simulate over ground walking (OGW) during treadmill training. OGW involves a cyclic acceleration-deceleration profile of pelvic velocity that contrasts with typical treadmill-driven walking (TDW), which constrains a person to walk at a preset constant speed. In this study, we investigated the effects of the proposed speed adaptation controller by analyzing the gait kinematics of UDW and TDW, which were compared to those of OGW at three pre-determined velocities.

**Methods:**

Ten healthy subjects were asked to walk in each mode (TDW, UDW, and OGW) at three pre-determined speeds (0.5 m/s, 1.0 m/s, and 1.5 m/s) with real time feedback provided through visual displays. Temporal-spatial gait data and 3D pelvic kinematics were analyzed and comparisons were made between UDW on a treadmill, TDW, and OGW.

**Results:**

The observed step length, cadence, and walk ratio defined as the ratio of stride length to cadence were not significantly different between UDW and TDW. Additionally, the average magnitude of pelvic acceleration peak values along the anterior-posterior direction for each step and the associated standard deviations (variability) were not significantly different between the two modalities. The differences between OGW and UDW and TDW were mainly in swing time and cadence, as have been reported previously. Also, step lengths between OGW and TDW were different for 0.5 m/s and 1.5 m/s gait velocities, and walk ratio between OGS and UDW was different for 1.0 m/s gait velocities.

**Conclusions:**

Our treadmill control scheme implements similar gait biomechanics of TDW, which has been used for repetitive gait training in a small and constrained space as well as controlled and safe environments. These results reveal that users can walk as stably during UDW as TDW and employ similar strategies to maintain walking speed in both UDW and TDW. Furthermore, since UDW can allow a user to actively participate in the virtual reality (VR) applications with variable walking velocity, it can induce more cognitive activities during the training with VR, which may enhance motor learning effects.

## Background

Body weight supported treadmill training (BWSTT) has shown its effectiveness in improving gait abilities of patients with neurological disorders such as stroke, spinal cord injury, Parkinson’s disease (PD), and traumatic brain injury (TBI) [1-6]. Some studies [7,8] found advantages of robot-aided treadmill training compared to BWSTT, while others found BWSTT to be more effective with larger variability [9,10]. Duschau-Wicke et. al [11] has inferred that robot-aided treadmill training is more effective for severely affected non-ambulatory patients while BWSTT may be more ideal for highly ambulatory patients. Although a recent large scale clinical trial on stroke rehabilitation reports that the outcome of BWSTT is not superior to the conventional therapy [12], BWSTT is still attractive since it provides a controlled and safe environment to patients, clinicians, and researchers. With advanced technology and greater understanding of recovery mechanisms, these might be made even more effective with specific design modifications that introduce greater intra-task variability and require greater cognitive engagement.

In recent studies, the combination of a virtual reality (VR) interface and a treadmill system produced synergistic benefits for gait rehabilitation [5,13-15]. Individuals with post-stroke hemi paresis in the VR treadmill groups improved their gait speed more than those who walked on the treadmill alone [13] as well as greater recovery in cognition and perception deficits [14]. Treadmill training with VR [5] is also feasible in PD and may significantly improve gait performance in more complex or cognitively challenging situations such as dual or multi-task conditions. A VR interface with a moving treadmill can simulate a variety of real world environments, thus motivating a patient to be more engaged in realistic and complex training [15].

However, previous instrumental gait rehabilitation protocols [3-6] were limited to constant walking velocity and could not provide velocity variability during training. Realistic walking should allow patients to voluntarily change walking speed, which is not only critical for patient safety but may also help patients participate more actively in cognitive tasks during VR training. Moreover, patients with hemi paresis may have time-varying gait patterns as well as asymmetric step lengths and durations. PD patients who may abruptly reduce walking speed due to freezing of gait (FOG) are at risk to fall off the treadmill belt if it is unable to respond to the sudden change. Thus, self-selected control of treadmill speed is required to provide more realistic walking conditions during VR interactions, adding variability, and greater potential of motor training while guaranteeing the safety of patients.

There are several studies that have implemented self-selected speed control of treadmills for gait rehabilitation. von Zitzewitz et al. [16] developed a voluntary speed adaptation controller of a treadmill with robotic gait orthosis, Lokomat, which can allow patient-cooperative control by measuring horizontal interaction forces through a mechanical tether connected to the trunk, similar to the Sarcos Tread port [17]. Koenig et al. [18] updated the algorithm to include speed adaptation for severe patients using swing leg forces. Even though mechanical tethers [16,17] can increase safety during patient training with fixed positioning, they may limit natural variability of walking in highly ambulatory patients due to motion constraints by the mechanical characteristics [19] of a tether or exoskeleton robot.

Other studies have focused mostly on body position feedback [20,21], aiming to provide natural walking without restricting human mobility. The feedback controls, which measure the positions of a body segment such as the pelvis and head, try to maintain the body position near a reference point. Similarly, a self-paced treadmill mounted onto a 6-degree-of-freedom motion platform with the CAREN (Computer Assisted Rehabilitation Environments, Motek Amsterdam) graphic system [22] also uses neutral positions for treadmill control with PID (proportional-integral-derivative) servo control. However, if a user rapidly changes walking speed, the feedback error (the distance between a body position and a reference point) increases quickly and generates a large inertial force. This can cause instability in a typical commercial treadmill with limited belt size. Souman et al. [23] combined a feedback controller with a dynamic observer to estimate pelvic velocity to implement realistic walking on a treadmill (6 meters long). However, the longer track is needed to allow a faster walking speed because faster walking causes larger anterior-posterior pelvic fluctuation [24], greater observer estimation error, and results in greater deviation between body position and a reference point. Recently, Feasel et. al [25] developed the integrated virtual environment rehabilitation treadmill (IVERT) system to estimate walking speed in asymmetric gait by using ground reaction forces measured from an instrumented treadmill and processed by a Kalman filter.

For rehabilitation purposes it is very important to guarantee stability of the treadmill controller even in the worst case scenarios such as sensor data loss, which often becomes an issue in real-time measurement [26]. The controller also should not disturb the patient’s intended walking patterns by inappropriate control actions when simulating OGW. Thus, the concept of a feed-forward control scheme [27] that estimates walking speed using specific gait parameters may be a good approach for gait rehabilitation to safely control speed of a treadmill without causing instability or unintended control actions. Even though step length and cadence have the strongest relationships with gait velocity among gait parameters [28], these existing temporal-spatial gait parameters are dependent on an individual’s leg length and unique walking characteristics. In addition, these parameters can only be measured after a single step is completed, which may cause delays in the real-time control. Similarly, average speed during one step can be measured by an inertial sensor [29] or motion tracker [30,31]. However, one study [32] did show that acceleration can be generated within a one-step period, which demonstrates the importance of fast detection. Recently, Yoon et al [33] proposed that the maximum swing foot velocity (MSFV) is linearly proportional to the average pelvic velocity during one step for OGW. This value can be updated within a half step period since it occurs in mid-swing period. Regardless of pelvic position, walking speed on a treadmill can be precisely estimated using swing foot velocity measurements, allowing walking speed to be safely and naturally controlled on a treadmill without affecting the user’s walking intention or causing instability of a treadmill controller. Based on the proposed speed estimation method, self-selected speed control of a treadmill can provide more natural and safer gait training. The speed adaptation controller can enable UDW in order to more closely simulate OGW, whereas a typical treadmill drives a person to walk at a preset constant speed.

In this paper, the speed estimation scheme is explained in detail and performance of the speed adaptation controller based on the proposed estimation scheme is objectively evaluated by analyzing the gait kinematics of UDW and TDW, which were compared to OGW at several pre-selected speeds. We compared and analyzed temporal and spatial gait parameters, as well as pelvic motion to obtain objective performance results.

## Methods

### Speed estimation

#### Speed estimation using swing foot motion

**Figure 1:**
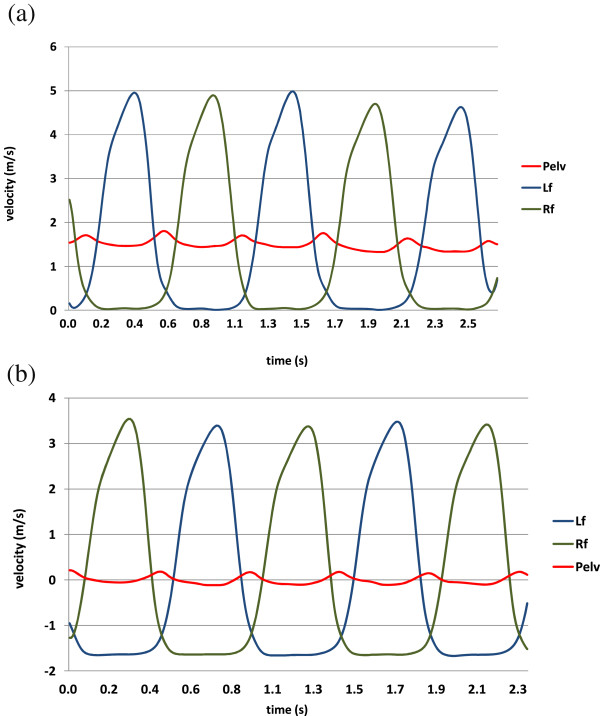
**Foot velocities along the forward walking direction during overground and treadmill walking.** The swing foot velocities are similar during overground and treadmill walking. Foot velocity during stance phase is zero in overground walking (Figure 1(**a**)) and negative on a treadmill, since it is caused by the treadmill speed (Figure 1(**b**)). It should be noted that magnitudes of sinusoidal trajectories in both cases are equal. (**a**) Foot and pelvis velocities (Overground) (**b**) Foot and pelvic velocities (Treadmill).

In this sub-section, we propose a novel method for detecting the user’s intention to change their walking speed by using MSFV. The mathematical relationship between MSFV and walking speed will be shown assuming that the velocity profile of the swing foot has the shape of a sinusoidal function (Figure 1). Swing foot velocity *V*_*sw*_ can then be represented as

(1) $$V_{\text{sw}}=V_{\text{mag}}\text{sin}\left(\frac{\pi }{T_{\text{sw}}}t\right),0\leq t\leq T_{\text{sw}}$$

where *V*_*mag*_ and *T*_*sw*_ represent the magnitude and the period of the sine function, respectively. The step length can be calculated by integrating (1) during the swing time (from left (or right) toe off to left (or right) heel strike) which is the same as *T*_*sw*_:

(2) $$S_{\text{sw}}=\overset{T_{\text{sw}}}{\underset{0}{\int }}V_{\text{sw}}dt=\overset{T_{\text{sw}}}{\underset{0}{\int }}V_{\text{mag}}sin\left(\frac{\pi }{T_{\text{sw}}}t\right)dt$$

By integrating and solving (2), the step length can be represented as follows:

(3) $$S_{\text{sw}}=2V_{\text{mag}}\frac{T_{\text{sw}}}{\pi }$$

By dividing (3) into the swing period, an average walking speed during the swing foot period can then be derived as follows:

(4) $$V_{sw,avg}=\frac{S_{\text{sw}}}{T_{\text{sw}}}=\frac{2V_{\text{mag}}}{\pi }$$

**Figure 2:**
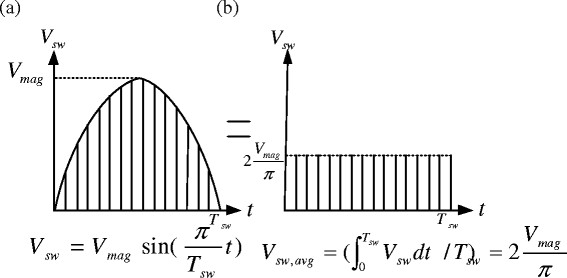
**Calculation of the average velocity by MSFV.** Swing foot velocity can be modelled as a sine function with period *T*_*sw*_ and magnitude *V*_*max*_; average walking speed during the swing foot period can then be derived as $V_{sw,avg}=\frac{2V_{\text{mag}}}{\pi }$. (**a**) Swing foot velocity trajectory (**b**) Average velocity of the swing foot.

**Figure 3:**
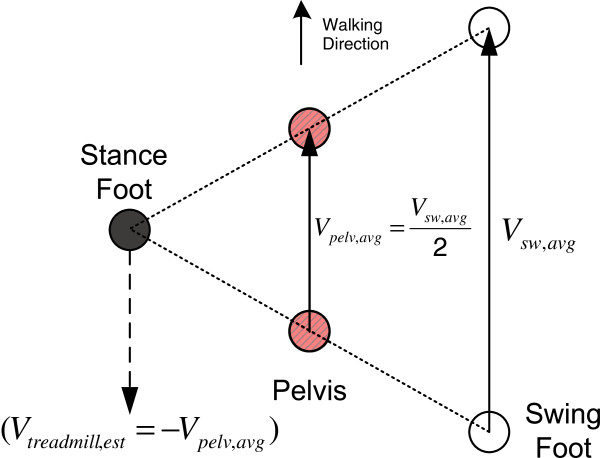
**Estimation of the pelvis velocity.** The average pelvic velocity should be half the swing foot velocity.

It should be noted that the resultant average velocity of the swing foot during the swing phase is a function of the MSFV (Figure 2(b) and Eq. (4)). In order to maintain balance of the human body during walking, the average pelvic velocity should be half the swing foot velocity, as shown in Figure 3. Thus, the average walking speed by using the pelvic (or body) velocity can be obtained as follows:

(5) $$V_{pelv,avg}=\frac{V_{sw,avg}}{2}=\frac{V_{\text{mag}}}{\pi }$$

**Figure 4:**
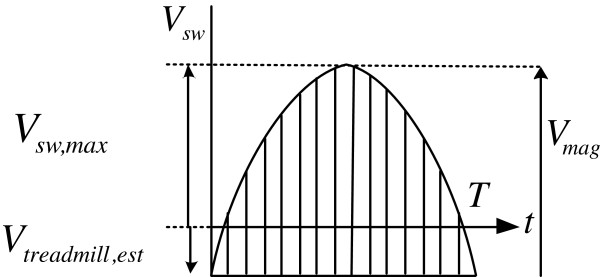
**Swing foot velocity on a treadmill.** The walking speed *V*_*treadmill,est*_ on a treadmill can be estimated as $-\frac{V_{sw,\max}}{\pi -1}$ by shifting the swing foot velocity trajectory *V*_*sw*_.

Similarly, the walking speed on a treadmill can be estimated by shifting the swing foot velocity trajectory, as shown in Figure 1 and Figure 4. The estimated treadmill speed, *V*_*treadmill,est*_, moves the treadmill at the same speed but opposite to the user’s walking direction, and can be represented by the negative value of the pelvic average speed as follows (also see Figure 3).

(6) $$V_{treadmill,est}=-V_{pel,avg}=-\frac{V_{\text{mag}}}{\pi }$$

On a treadmill, the magnitude of the sinusoidal velocity of the swing foot can be expressed as the following (Figure 4):

(7) $$V_{\text{mag}}=V_{sw,\max}-V_{treadmill,est}$$

where *V*_*sw,max*_ is the measured maximum foot velocity during treadmill walking. Thus, by inserting (7) into (6), and arranging the equations with respect to *V*_*treadmill,est*_, the treadmill speed estimation can be obtained as follows:

(8) $$V_{treadmill,est}=-\frac{V_{sw,\max}}{\pi -1}$$

It should be noted that *V*_*sw,max*_ is used to estimate the walking speed on a treadmill, while *V*_*mag*_ is used for speed estimation in OGW.

Equations (5) and (8) show important characteristics in walking speed estimation using temporal and distance parameters. Speed can be directly estimated without normalizing for leg length or height and does not strongly depend on individual characteristics such as step length and cadence. The index can be detected during the first half of the swing phase, which will allow for speed adaptation within a half step period. Thus, the speed of the treadmill can be updated immediately while the other foot (stance foot) is on the treadmill. Quicker estimation can make the treadmill react more rapidly to user’s intention for velocity change. This feature may also be appropriate for abrupt changes such as FOG in PD to quickly update treadmill speed, which should help prevent falling off the treadmill [34]. In addition, velocity estimation of independent swing foot trajectories may be applicable for individuals with hemi paresis who have asymmetric step lengths and durations.

#### Verification of the speed estimation scheme

**Figure 5:**
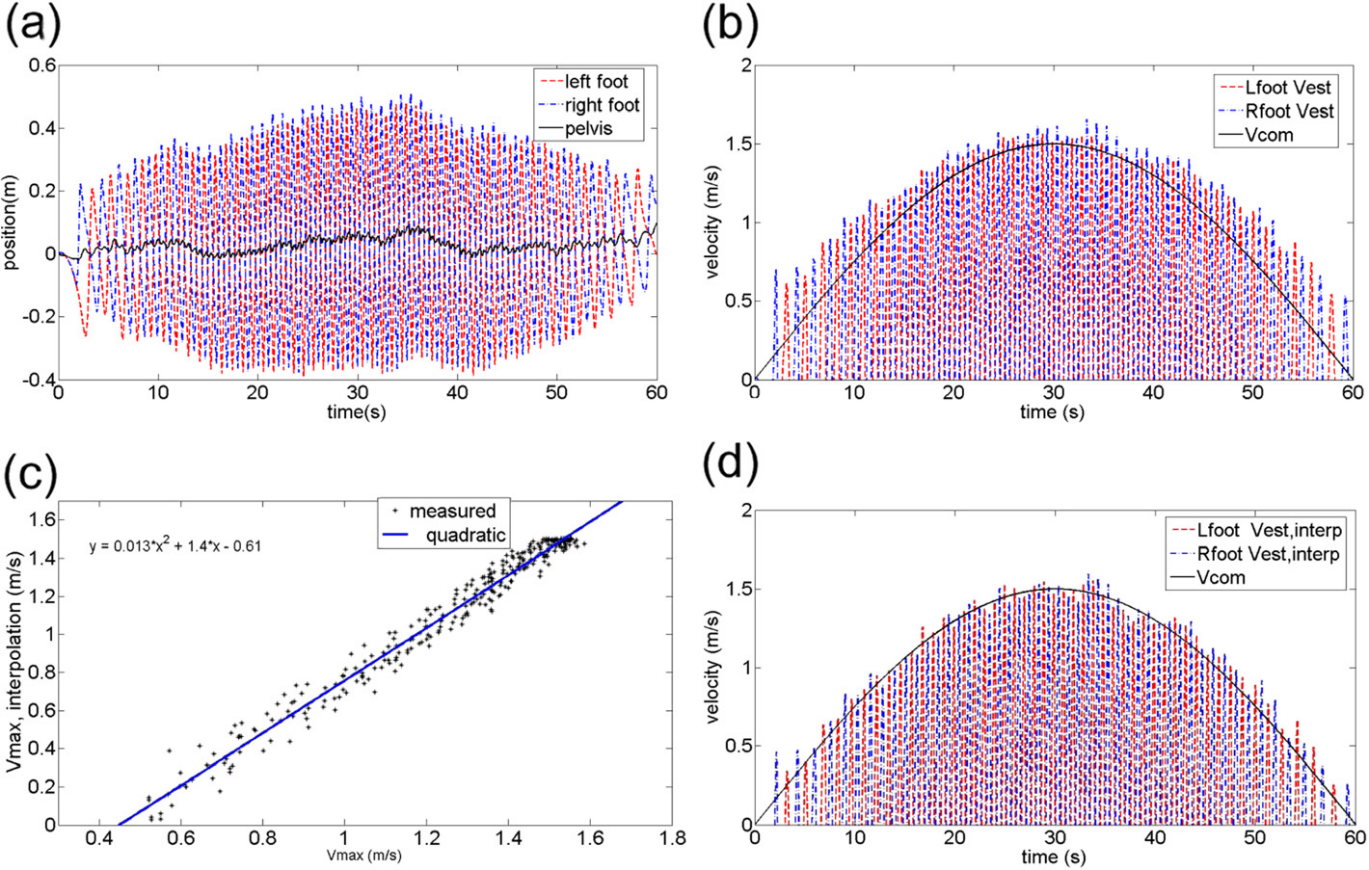
**2nd order interpolation for walking velocity estimation.** 2^nd^ order interpolation results for walking velocity estimation with one exemplary subject. (**a**) Foot and pelvis positions (**b**) Foot velocity vs. walking velocity (**c**) Second-order interpolation (**d**) Foot velocity after interpolation for error compensation vs. walking velocity.

In order to verify the speed estimation scheme, subjects walked on a treadmill that was controlled to follow a predefined velocity profile generated by a half period of a sinusoidal function with a magnitude of 1.5 m/s and a period of 60 s (the solid line in Figure 5(b)). The estimated speed (the peak points in Figure 5(b)) calculated by (8) matches the real treadmill speed (solid line in Figure 5(b)) relatively well. In Figure 5(b), the estimated speed (peak points) matches well at walking speeds of approximately 1.5 m/s; however, the deviation from the treadmill speed (actual walking speed) becomes larger at lower speeds. This may be due to an increase in the double support phase at lower speeds, which caused faster swing foot movement. Since there is a deviation in strategy at lower speeds, we applied a second-order interpolation based on Figure 5(b) to compensate for the deviation. Figure 5(c) shows the relationship between MSFV and treadmill speed (actual walking speed), and the second-order interpolation function that mapped the MSFV to the walking speed. The error at lower speeds was reduced after the compensation (Figure 5(d)). The 2nd order interpolation can compensate modeling errors between real swing and sinusoidal trajectories and can minimize the errors of velocity estimation for each subject.

### Speed adaptation control

#### Speed adaptation control scheme based on swing foot motions

**Figure 6:**
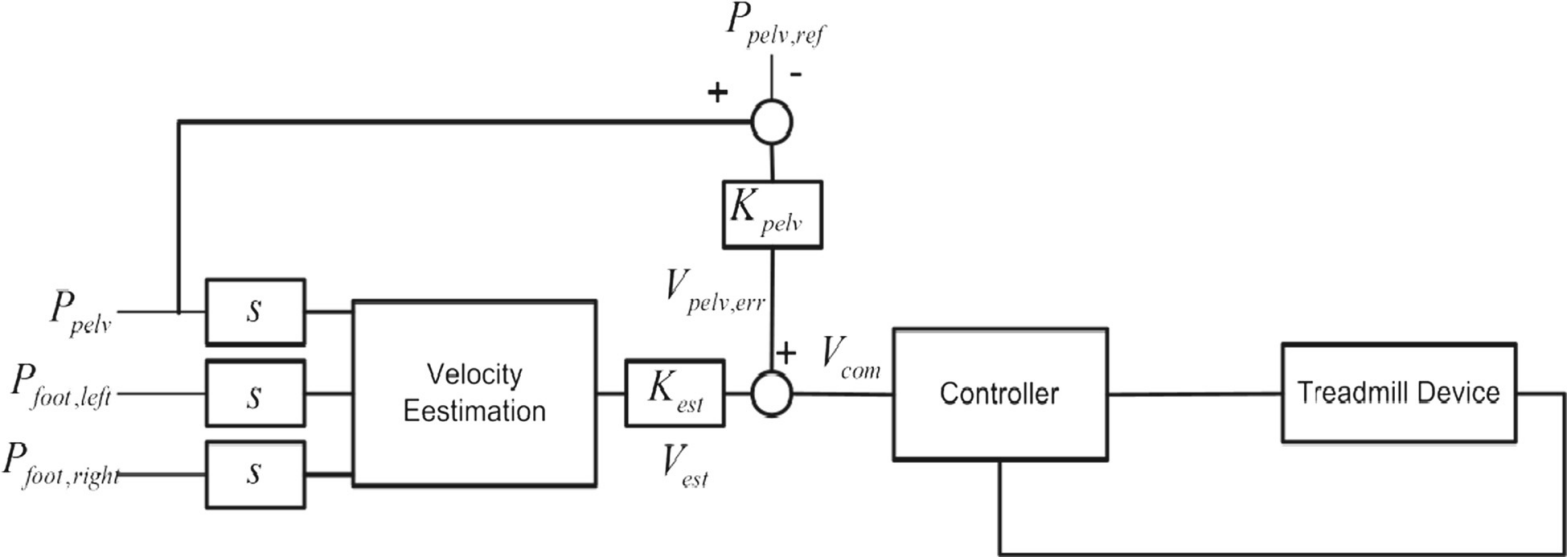
**Block diagram of the treadmill speed control algorithm**.

Self-selected speed control is constructed by combining the proposed speed estimation method as a feed-forward controller and a feedback controller with anterior-posterior pelvic motion as a supplement to the feed-forward controller:

(9) $$V_{treadmill,com}=V_{treadmill,est}^{\text{int}erp}+V_{pelv,err}$$

where $V_{treadmill,est}^{\text{int}erp}$ is the estimated walking speed and *V*_*pelv,err*_ is the pelvic feedback control term. The estimated walking speed based on the MSFV could be obtained from (8) after the second-order interpolation:

(10) $$V_{treadmill,est}^{\text{int}erp}=K_{\mathrm{est}}\left(ax^{2}+bx+c\right)$$

where *x* = *V*_*treadmill,est*_ and *K*_*est*_ is the proportional gain of the estimated walking speed. It should be noted that we are calculating foot velocity with respect to pelvis. Therefore, pelvic position is needed to predict the estimated walking speed in Figure 6. The pelvic feedback control was defined as follows:

(11) $$V_{pelv,err}=K_{pelv,err}\left(P_{\mathrm{pelv}}-P_{\mathrm{ref}}\right)$$

where *P*_*pelv*_ and *P*_*ref*_ denote the measured pelvis position and reference pelvis position, and *K*_*pelv,err*_ represents a proportional feedback gain. $V_{treadmill,est}^{\text{int}erp}$ corresponds to the feed-forward term that estimates the user’s walking speed while *V*_*pelv,err*_, the feedback term, maintains the user near *P*_*ref*_ . The control command (9) for speed adaptation will be sent to a belt speed controller that is composed of a PID control. Figure 6 shows the overall control structure of the proposed algorithm.

The proposed controller can maintain the feedback proportional term within 10% during even abrupt walking speed changes [33], and does not cause significant overshoot or instability problems due to the precise speed estimation by the proposed speed estimation scheme. As the simple feedback gain for neutral positioning was decreased and the feed-forward gain for velocity estimation was increased, we observed that variability in the pelvic acceleration decreased. This may show a reduced alteration of natural walking patterns on a treadmill [33]. Thus, the proposed controller enables UDW in order to more closely simulate OGW while minimizing any alterations of the user’s walking patterns since speed estimates on a treadmill could precisely predict a user’s walking intension.

#### Detecting phase transition and user’s stop intention from the foot velocity measurement

The proposed controller utilizes the double stance period to rapidly detect the stop intentions of the user, which is one of the key advantages of using foot motion. Stance and swing phases are determined by the swing foot velocity threshold, which was set at 0.1 m/s. The swing foot velocity during normal walking is approximately 4-6 m/s, which is much greater than the threshold value; therefore, the phase detection has a maximum delay of 2 ms. The stance phases of each foot are detected separately, and hence the double stance phase can also be precisely detected based on the phases of the two feet. Then, the user’s intention to stop walking can be detected by utilizing the length of the double stance phase. If a double stance phase becomes 20% longer than that of the previous step, the controller considers it as the user’s intention to stop walking. The threshold was determined based on preliminary tests with healthy volunteers during the controller development. When the threshold was higher than 20%, it caused time delay of stop detection, while at lower than 20%, it might misinterpret normal walking as stop intention. The time to detect the threshold depends on the walking speed. The maximum time delay to detect the intention to stop is approximately 25 ms for 0.5-1.5 m/s walking speed. Subsequent to the stop detection, the treadmill moves the user toward the neutral position (typically set as center) of a treadmill belt at a slow speed. Then, the treadmill controller is set ready for another walking trial.

### Experimental design

A gait analysis with temporal-spatial parameters was performed with respect to normal walking conditions on the treadmill, to obtain objective performance results. For this purpose, we classified the walking modalities on the treadmill into UDW and TDW. UDW allows a user to voluntarily change their walking speed on the treadmill, and the treadmill follows their lead. In contrast, TDW sets a pre-determined speed and users have to adjust their speed initially to keep up with the treadmill and after a while they develop a regular fixed pattern to follow the treadmill. OGW is also performed to provide standard values for natural walking compared to UDW and TDW.

#### Measurement and control setup

**Figure 7:**
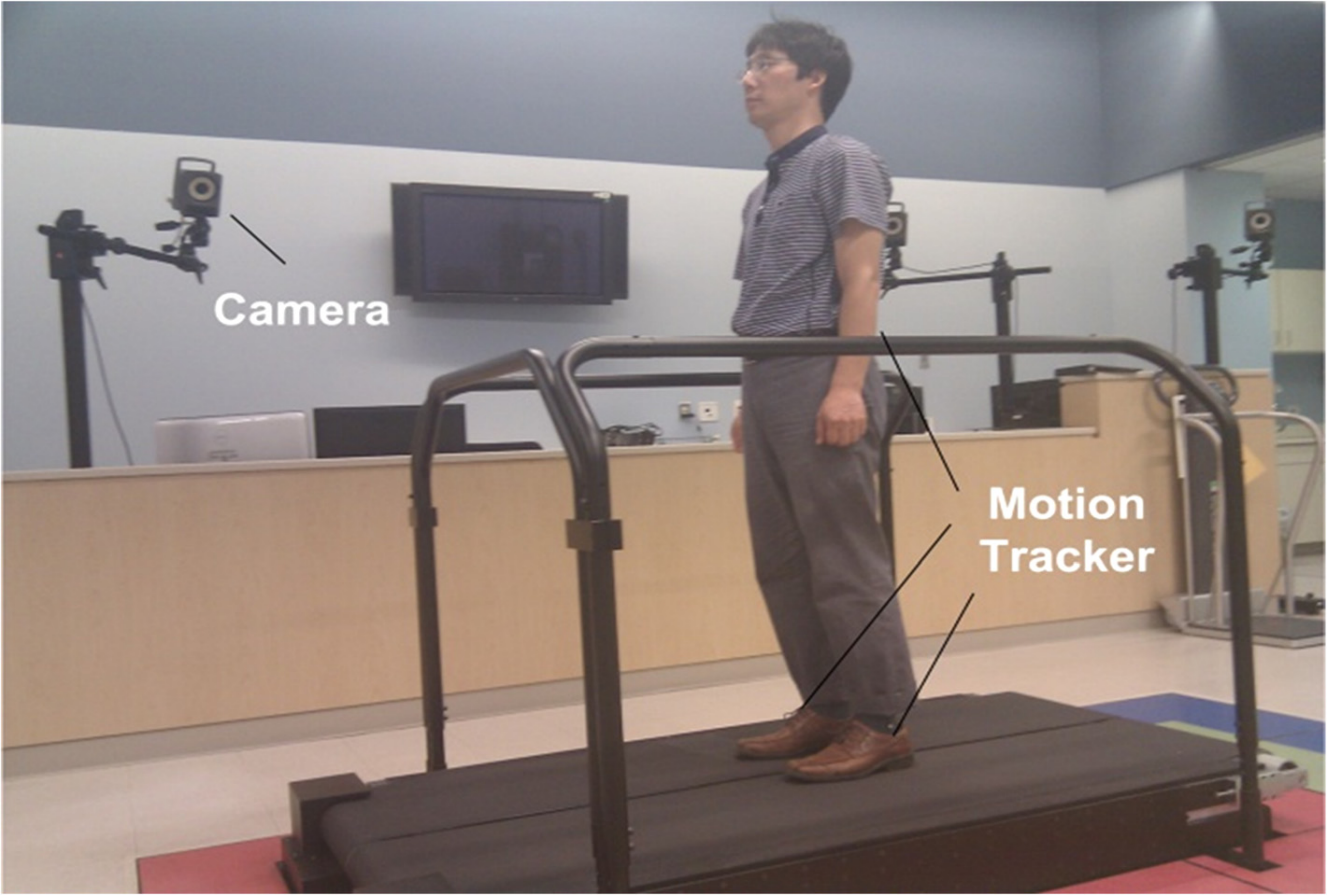
**Measurement setup.** The user’s pelvic and foot motions were captured by the VICON motion capture system at a sampling rate of 120 Hz.

The user’s pelvic and foot motions during OGW and treadmill walking were captured by the Vicon motion capture system (Vicon Motion Systems Inc., Centennial CO, USA). Two markers were attached on the lateral side of each foot at a distance of 2 cm below the ankle joint (see Figure 7). An additional two markers were placed on the skin overlying the posterior superior iliac spines for pelvis tracking as shown in a short Additional file 1. For patients, we can attach pelvis markers to adequate harness locations corresponding to the posterior superior iliac spines of a patient. The positions of the markers were captured at a sampling rate of 120 Hz.

For treadmill tests, subjects walked on a split-belt treadmill (Bertec Co., Columbus OH, USA). Although each belt of the treadmill was designed to move independently, the two belts moved in unison. The size of the treadmill was 1.5 (L) x 0.8 (W) m^2^ and had a maximum speed of 6.0 m/s and a maximum acceleration of 6.0 m/s^2^. Speed commands to the treadmill were provided through a TCP-IP connection by a custom-written C++ program that sent out speed commands at 120 Hz. To prevent excessive movements of the treadmill and to guarantee the safety of the users for UDW, the feedback and feed-forward gains were fixed at K_*pelv*_ = 0.5 and K_*est*_ = 1, which had showed the best performance during pilot testing. For safety purposes, hand rails were installed on the front and two sides of the treadmill so that the participants could grab the bar if they needed; acceleration of the treadmill was limited to 1 m/s^2^ . For data analysis, only data collected while users did not grab the bar were used.

#### Subjects

Ten healthy volunteers (6 men, 4 women) ranging in age from 18 to 38 years (27.6 +/- 7.0), height 160-174 cm (167.2 +/- 4.8), participated in this study. All participants signed informed consent approved by the Institutional Review Board at the National Institutes of Health prior to the experiment.

#### Protocol

For OGW, the subjects were instructed to walk on level ground at predefined speeds (0.5 m/s, 1.0 m/s, 1.5 m/s) while their walking speed and target speed were displayed on a monitor screen by using a custom LABVIEW program. The subjects practiced several times for adjusting their walking to the target walking speeds. About a 5 m distance was used to measure gait patterns with steady-state pelvic velocity [35]. The gait patterns during acceleration and deceleration periods were deleted through post processing. Then, the subjects walked passively on the treadmill with the predefined sinusoidal velocity trajectory as shown in Figure 5(b). The walking data were stored in a custom-developed software program and analyzed for interpolation acquisition of the MSFV with MATLAB 7.0 (Mathworks, Natick MA, USA). After interpolation, the subjects were asked to walk at the three pre-determined speeds on the treadmill. The same speed profile was tested under the two walking modalities (i.e., UDW and TDW) in a randomized order. The data were collected for 20 seconds for each condition. For each UDW, TDW, and OGW, the subjects watched a monitor for visual feedback during walking so that the subject could maintain the desired speed. After completing a few practice trials for each modality, position and velocity data from the four markers were collected.

#### Data analysis

The parameters representing spatial and temporal gait and pelvic motion were analyzed, including the average step length, cadence, walk ratio (WR), and peak-to-peak pelvic acceleration. The step length was obtained by calculating 1) the time between the positive peaks of the left and right feet trajectories and 2) the corresponding distance traveled by the treadmill during the time, which represents approximately the distance between the ipsilateral and the contralateral heel at each heel contact. The average step length was then calculated at three speeds (0.5 m/s, 1.0 m/s, and 1.5 m/s). Cadence was calculated based on the number of steps per minute. The walk ratio [36] represents the relationship between the amplitude and frequency of rhythmic leg movements during walking, and was calculated as the average step length divided by the cadence [37]. For pelvic motions, the average peak acceleration and standard deviation were used to provide an average magnitude and variability of acceleration patterns. For speed estimations, experiments were conducted for each subject on the treadmill with the positive predefined speed profile, and the data collected for the speed estimation errors were averaged to evaluate the overall speed estimation performance.

In the statistical analysis, an ANCOVA (Analysis of Covariance) with velocity as a covariate was performed to test for significant differences across UDW, TDW and OGW at 0.5 m/s, 1.0 m/s, 1.5 m/s. The effect of velocity differences was removed by considering velocity as a covariate because temporal-spatial parameters are dependent on walking velocity, and walking velocity did not perfectly match the target speed in UDW and OGW. A one-factor balanced analysis of variance (ANOVA) was used to calculate other temporal-spatial gait parameters and parameters of pelvis motion as long as the speeds do not affect the statistical differences by the ANCOVA analysis. Statistical analyses were performed using the statistical analysis software SPSS v.10.0. Statistical significance was determined as a P-value of less than 0.05.

## Results

### Speed estimation results

**Figure 8:**
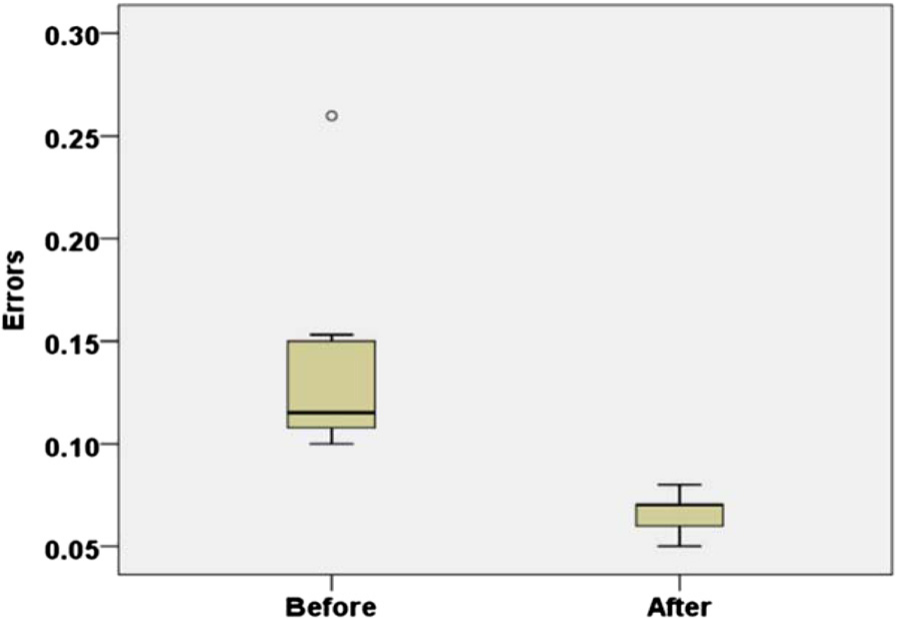
**Speed estimation error before/after interpolation.** After the proposed second-order interpolation, the speed estimation errors dropped to 0.0663 m/s and the standard deviations of the average estimation errors were also reduced to 0.01 (box-whisker plot).

Figure 8 shows the mean distributions of the speed estimation error for the ten subjects before and after interpolation. For the speed estimation errors of Figure 8, the errors between the estimated speed (the peak points in Figure 5(b)) and the real treadmill speed in Figure 5(b) are used for before section results of Figure 8, while the errors between the interpolated estimated speed and the real treadmill speed at Figure 5(d) are used for after section results of Figure 8. The treadmill speed was used for the standard of the speed estimation, since pelvic velocities on a treadmill are small enough to be neglected and a user’s walking speeds on a treadmill can be considered to treadmill speeds. The original speed estimation error was initially an average of 0.1354 m/s for the subject trials. In contrast, after implementing the proposed second-order interpolation during the process, the speed estimation errors dropped to 0.0663 m/s, indicating a 51% reduction. With the interpolation, the standard deviations of the average estimation errors were also reduced to 0.01, which shows that the estimation errors were not very dependent on individual subject characteristics since the SD (standard deviation) represents the individual variability of the estimation errors [38].

#### Trends in spatial and temporal gait parameters

**Figure 9:**
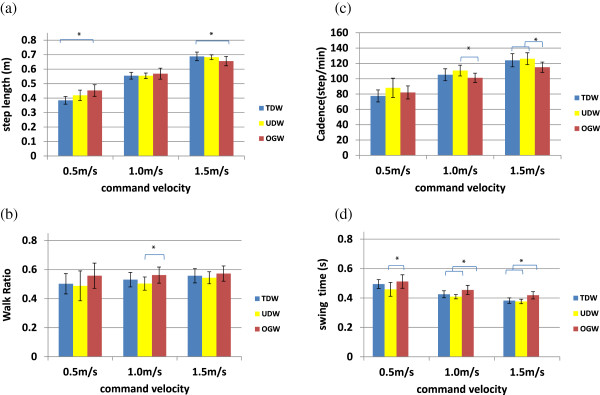
**Effect of walking speed on temporal-spatial gait parameters.** Temporal-spatial gait parameters between UDW and TDW are not significantly different for all gait speeds, while there was significant difference between OGW and UDW/TDW. (**a**) Step length (**b**) Walk ratio (**c**) Cadence (**d**) Swing time. OGW vs. UDW: cadence at 1.0 m/s (p = 0.02) and 1.5 m/s (p = 0.01), swing time at 0.5 m/s (p = 0.02), 1.0 m/s (p = 0.001), and 1.5 m/s (p = 0.001), and walk ratio at 1.0 m/s (p = 0.03). OGW vs. TDW: cadence at 1.5 m/s (p = 0.04), swing time at 1.0 m/s (p = 0.03) and 1.5 m/s (p = 0.001), step length at 0.5 m/s (p = 0.001) and 1.5 m/s (p = 0.03).

When the subjects were instructed to walk in each of the three modalities, the step length and cadence linearly increased with respect to the walking speed, whereas the walk ratio had relatively small variations with respect to the walking speed. Figure 9 shows the comparative results of the three walking modalities for the temporal-spatial gait parameters. The average speed of UDW was slightly greater than TDW which explains slightly increased cadence in UDW at the same instructed speed (0.62 (±0.08) at 0.5 m/s, 1.08 (±0.08) at 1.0 m/s, 1.53 (±0.11) at 1.5 m/s). Although all subjects tried to keep their walking speeds at 0.5 m/s, 1.0 m/s, and 1.5 m/s as directed, the actual walking speed in OGW was slightly but not significantly higher than the instructed speed at 0.5 m/s and was slightly but not significantly slower at 1.5 m/s (0.67 (±0.08) at 0.5 m/s, 1.02 (±0.08) at 1.0 m/s, 1.34 (±0.09) at 1.5 m/s). There was no significant effect of the walking modalities on any of the temporal-spatial gait parameters after adjusting for differences in walking velocity. The overall results suggest that the temporal-spatial gait parameters between UDW and TDW are not significantly different for all gait velocities.

#### Pelvis motion

**Figure 10:**
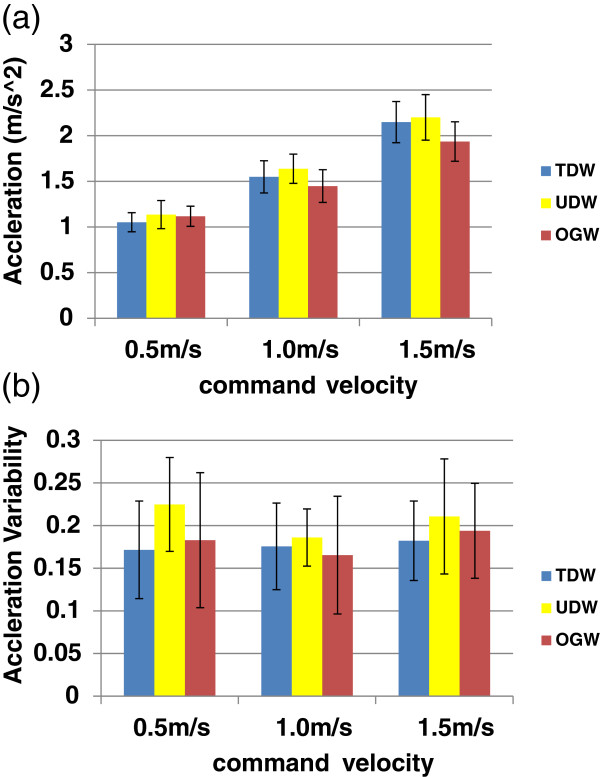
**Effect of walking speed on pelvis motion.** For the overall comparisons of the three modalities, the average total magnitude and variability for the pelvic acceleration did not differ. (**a**) Average peak magnitude of pelvic acceleration (**b**) Variability of pelvic acceleration.

As shown in Figure 10, for three modalities, the average peak magnitude of the pelvic acceleration increased with increasing walking speed, while the acceleration variability remained constant regardless of the walking speed. For the overall comparisons of the three modalities, the average peak magnitude and variability of the pelvic acceleration did not differ across the modalities. There was no significant effect of the walking modalities on any of the parameters of pelvic motions after controlling for walking velocity.

## Discussion

### Walking speed estimation

This research has proposed a new scheme to estimate walking speed on level ground and a treadmill based on the MSFV. Precise walking speed estimations for treadmills will be essential to develop an adequate automatic speed adaptation controller without disturbing the user’s gait patterns. The proposed walking speed estimation technique can easily and rapidly detect walking intentions within a half step. This contrasts sharply with the estimation using other gait parameters such as step length or cadence [27]. In OGW, pelvic velocity along the forward walking direction represents walking speed; however, in treadmill walking, the pelvis velocity fluctuates around zero as shown in the Figure 1(b) which does not represent walking speed. For this reason, we considered one degree-of-freedom (1 DOF) foot motion along the anterior-posterior direction for walking speed estimation. Based on our experiments, we determined that the MSFV was linearly proportional to the walking speed during treadmill walking. The COM will shift closer to the stance foot, especially when the walking speed is slower [39], thus, the estimation errors may become larger at slower speed as shown in Figure 5(b) since the pelvis (COM) is not situated equally between the stance and swing feet. Compensation for speed estimation errors was made for slow walking speed using a second-order interpolation function, which could be acquired quickly using a TDW experiment.

A linear regression analysis of the relationship between walking speed and related kinetic and kinematic parameters was presented by Stansfield et al. [28] for normal children. In their results, the R^2^ values were highest in cadence (R^2^ = 0.53) and step length (R^2^ = 0.53). When linear regression analysis was performed on the values from individual subjects, good fits were obtained for the MSFV (average R^2^ =0.95, R^2^ > 0.9 in all cases) and step length (average R^2^ =0.94, R^2^ > 0.9 in 9 of 10 cases) versus walking speed, while the relations between swing time and walking speed were less linear (average R^2^ =0.64, R^2^ > 0.7 in 7 of 10 cases). This implies that the MSFV is highly linear with respect to walking speed and has less variation with respect to different users than other existing temporal-spatial gait parameters.

During our experiments, we discovered that individual interpolation for each subject could reduce the speed estimation error and minimize the effect of individual walking characteristics. We incorporated interpolation parameters of each subject for UDW which did not add significant time to the whole protocol since it took less than five minutes, including three minutes of treadmill walking at the sinusoidal velocity trajectories. Even though the procedure is short, this interpolation procedure could be considered as one of the limitations for treadmill control compared to other methods [20-23] that do not require an extra trial. For obtaining interpolation parameters, the magnitudes of the sinusoidal trajectories can be set below their maximum walking speed. For patients, magnitude of sinusoidal trajectories can be set by the fastest walking velocity measured during the level ground assessment. We have only tested PD patients so far and their max walking speed ranged from 0.1 m/s to 1.5 m/s depending on degree of impairment [40].

Moreover, independent interpolation parameters for speed estimation at each foot can provide adequate speed update of a treadmill for patients with asymmetric gait patterns such as patients with hemiplegic stroke, cerebral palsy, and unilateral amputees. In order to avoid the different velocity update of left and right steps due to asymmetric gait patterns, we applied the average velocity of left and right feet to the desired treadmill velocity, which can minimize the belts jerk and keep the user stay within belts during walking. A weighted moving average (WMA) can be applied to patients, depending on impairment degree of patients. In addition, a separate speed control scheme [25] of a treadmill with independent belts may work better for stroke patients who have apparently different swing foot velocities.

If the rapid accelerations/decelerations occurred before the COM passed the stance foot (midstance), the opponent swing leg can reach the maximum speed at the mid swing phase to keep the balance of the COM with the assumption that the pelvis (COM) is situated equally between the stance and swing feet as shown in Figure 3. In those cases, the suggested control scheme can handle the rapid acceleration/deceleration with adequate velocity estimations. However, the gait patterns would be destabilized if the subject has a sudden stop or has rapid acceleration after the COM passed the stance foot (midstance). Based on the data from healthy volunteers, 20% increase in double stance phase was set as the threshold value to detect the intention to stop; however, this threshold value will need to be increased for patients who walk at slower speeds and whose double stance phase might be more variable.

For safety purposes, we inserted a safety algorithm to the treadmill controller, which replaces the current marker values to the pervious marker values if the marker values become almost zero due to the markers being blocked. This algorithm prevents the control command from being abruptly changed and causing instability. Since an instrumented treadmill was used in the study, we could have utilized ground reaction forces to control for treadmill speed [25]. We chose not to use kinetic data because our goal was to develop a method that can be implemented with typical treadmills widely used in clinical settings for BWSTT. Although we measured foot velocity from the motion capture system, this can be easily replaced by wireless accelerometers which are widely available as a result of recent developments in wireless sensor technology [41,42]. If a traditional treadmill is to be modified for the user controlled walking mode, commercially available treadmills will need to be able to accommodate with control inputs from user walking. Thus, this standard interface for treadmill control will be needed to move forward with user controlled treadmills.

### User studies

During user studies, all subjects were able to walk on the treadmill with UDW, even though they had no previous experience on treadmill walking under speed adaptation control. The temporal-spatial gait parameters such as step length, cadence, walk ratio, and pelvic motions were not significantly different for UDW and TDW. Swing time and cadence between OGW and TDW (or UDW) were significantly different. For pelvis motions, the peak average magnitude of the pelvic acceleration and its variability were not significantly different between OGW, and TDW (or UDW).

The differences for swing time and cadence between TDW and OGW were also reported in other studies. From comparisons between OGW and TDW at preferred walking speed, Lee and Hidler [1] showed that swing time at TDW is shorter than that of OGW, and Wass et. al [2] showed that higher initial cadence at TDW is similar to OGW after sufficient accommodation time on the treadmill. Even though the walking in OGW were slightly different from those of TDW in terms of temporal-spatial parameters, treadmill training for stroke patients was shown to improve spatial and temporal gait characteristics more effectively than walking outdoors [43].

A study by Hylton et al. [37] found that in order to stabilize walking, users changed their stepping patterns to maintain velocity by adapting cadence, stride length, walk ratio, and acceleration variability. Likewise, if UDW disturbed users’ stable walking, they would have changed their stepping pattern to maintain a target speed on a treadmill. However, our results demonstrate that the temporal-spatial parameters and pelvic acceleration in UDW and TDW were not significantly different. This suggests that users can walk during UDW as stably as TDW and have similar stepping pattern to maintain walking speed in both UDW and TDW.

The proposed speed adaptation algorithm simulates natural walking while causing users to attempt to voluntarily change (or maintain) walking speed. This may lead to a more enhanced sensation of total immersion walking using the proposed UDW during walking navigation in virtual environments. Recent studies [9,10,12] on BWSTT reported that the effectiveness was not greater than that of intensive conventional therapy although it provided a convenient training environment. After an initial accommodation period, BWSTT may provide a too regular rhythm, and a minimal amount of variability in practice due to a fixed belt speed. Therefore, the main advantage of the proposed controller is that it can allow patients to practice reacting to variable environments. Variability in training may bring in greater training effect [44]. In addition, it can be used as an assessment tool since it can simulate OGW where patients respond to environmental perturbations. For example, FOG could perhaps be elicited on a treadmill by manipulating the external environment to simulate natural conditions using VR while the proposed controller allowed users to walk as naturally as OGW [40].

The data analysis performed in our study also proposes methodology to evaluate the performance of a self-selected treadmill controller in a quantitative way. Previous studies evaluated the performance of self-selected treadmill controllers by comparing questionnaires between two different experimental setups [16] or different control gains [23]. This study compared statistical differences in temporal-spatial gait parameters between different modalities in more objective ways, which can demonstrate whether the suggested controller affects walking patterns of a user on a treadmill.

## Conclusions

We proposed a novel walking speed estimation technique based on swing foot velocity measurements to precisely and rapidly predict anterior-posterior pelvic velocity within a half step and have developed a self-selected treadmill speed controller with the novel speed estimation scheme. Our treadmill control scheme provides nonintrusive and safe self-selected speed control, which may be adequate for highly ambulatory patients with neurologic disorders such as mild TBI, mild stroke, cerebral palsy, PD [40], and others with gait limitations. More importantly, it implements similar gait biomechanics as TDW, which has been widely used in gait rehabilitation. It also promotes cognitive engagement during the training, which may produce a greater learning effect. For future development, inertial sensors for measuring swing foot and pelvis motions in a more compact and simple manner will be used to replace the optical motion markers for better portability and availability. It is also necessary to perform further systematic analysis to study the responses of a self-selected treadmill controller during rapid accelerations/decelerations, and to perform fair performance comparisons with other self-selected treadmill controllers. Clinical trials will be performed to determine the clinical effects of UDW as an alternative intervention to TDW protocols.

## Abbreviations

VR: Virtual Reality; TT: Treadmill Training; UDW: User-Driven treadmill Walking; OGW: Over ground Walking; TDW: Treadmill-Driven Walking; BWSTT: Body Weight Supported Treadmill Training; PD: Parkinson’s Disease; TBI: Traumatic Brain Injury; FOG: Freezing Of Gait; CAREN: Computer Assisted Rehabilitation Environment); PID: Proportional Integral Derivative; IVERT: Integrated Virtual Environment Rehabilitation Treadmill); MSFV: Maximum Swing Foot Velocity; WR: Walk Ratio; ANCOVA: Analysis of COVAriance; ANOVA: Analysis Of Variance; SD: Standard Deviation.

## Competing interests

The authors declare that they have no competing interests.

## Authors' contributions

JY performed the measurements of all patients, data analysis, and drafted the manuscript. HSP managed whole project, participated in the measurements of all patients, the design and coordination of the study, and assisted with drafting the manuscript. DLD managed the clinical protocol and assisted with drafting the manuscript. All authors read and approved the final manuscript.

## Supplementary Material

Additional file 1 **Walking behavior with UDW.** A short movie file is attached as an electronic supplementary material showing a subject walking with the proposed treadmill controller (UDW). Click here for file
